# Aquaporin 1, Aquaporin 8, and Aquaporin 9 Expressions in Malignant Melanoma: A Possible Correlation with Prognosis and Clinical Outcome

**DOI:** 10.3390/jcm12227137

**Published:** 2023-11-16

**Authors:** Lara Camillo, Elia Esposto, Laura Cristina Gironi, Chiara Airoldi, Shahd Abdullah Alhamed, Renzo Luciano Boldorini, Elisa Zavattaro, Paola Savoia

**Affiliations:** 1 Department of Health Sciences, University of Eastern Piedmont, 28100 Novara, Italy; lara.camillo@uniupo.it (L.C.); 20047737@uniupo.it (S.A.A.); renzo.boldorini@med.uniupo.it (R.L.B.); paola.savoia@med.uniupo.it (P.S.); 2 AOU Maggiore della Carità, 28100 Novara, Italy; elia.esposto@maggioreosp.novara.it (E.E.); gironi.laura@gmail.com (L.C.G.); 3 Department of Translational Medicine, University of Eastern Piedmont, 28100 Novara, Italy; chiara.airoldi@uniupo.it

**Keywords:** AQP, malignant melanoma, nodular melanoma, BRAF mutation, superficial spreading melanoma, melanocytes, Breslow index, mitotic index

## Abstract

Aquaporins (AQPs) are small transmembrane proteins able to facilitate the passive transport of water and small molecules throughout cells. Several studies have demonstrated that modulation of AQPs’ expression contributes to cancer development and progression. However, to date, very little is known about their involvement in malignant melanoma (MM) progression. In this retrospective observational study, we evaluated the correlation between AQP1, -8, and -9 expression and the clinical outcomes of 58 patients diagnosed with MM from 2014 to 2016, of which 14 were diagnosed as nodular melanoma (NM) and 44 as superficial spreading melanoma (SSM). In general, we found that AQPs were more highly expressed in SSM than NM, suggesting a potential correlation with prognosis. While analyzing the expression of each AQP, we discovered that AQP1 was associated with a specific body site and low mitotic index, AQP8 with a negative sentinel lymph node, and AQP9 with the Breslow thickness and lack of ulcerations. Together with the survival analysis performed in this study, our results suggest that the expression of AQP1, -8, and -9 could be correlated with a better prognosis for malignant melanoma.

## 1. Introduction

Malignant melanoma (MM) represents the most aggressive type of skin tumor and plenty of studies have pointed out the importance of understanding the pathophysiological processes associated with its metastatic behavior. Addressing these mechanisms could, in fact, contribute to establishing new therapeutic approaches and improve the outcomes of many hopeless melanoma patients. Aquaporins (AQPs) are a family of small transmembrane proteins of about 24–30 kDa, widely expressed in the animal and plant kingdoms, which facilitate water flow throughout cells [1]. The existence of water channel proteins has been predicted for a long time but was not identified until the pioneering discovery of AQP1 by Peter Agree and colleagues, which confirmed its water-selectivity, suggesting its fundamental importance for transmembrane or transcellular water transport into tissues [2]. Subsequent studies showed that AQPs can also facilitate the transport of other small and uncharged molecules, such as glycerol, urea, small gases, and hydrogen peroxide [3]. According to their molecular structure, AQPs are subdivided into three families: (i) classical AQP (water selective), including AQP0, AQP1, AQP2, AQP4, AQP5, AQP6, and AQP8; (ii) aquaglyceroporins (glycerol channel), including AQP3, AQP7, AQP9, and AQP10 [4]; and (iii) superaquaporins (AQP11 and AQP12), permeable to ions and gases (such as O_2_, CO_2_, and NO) [2]; these latter AQPs have only 20% homology with the classic AQPs and are expressed only in the cytoplasm. Besides the regulation of water homeostasis, AQPs are involved in many cellular processes and have been widely associated with the development of several tumors, including skin cancers [5]. Indeed, AQP1 is involved in angiogenesis, cell migration, and cell growth [6] and regulates the exchange of water between blood and the dermis to maintain the correct state of hydration of the skin [7]. However, alteration of AQP1 expression has been associated with different tumors [8], including non-melanoma skin cancer (NMSC) and melanoma, though its role is still not completely clear. Indeed, in vivo studies conducted on a mouse model by Nicchia et al. [9] showed that AQP1 is associated with poor prognosis in MM, while Imredi et al. [10] showed, for the first time, that the overexpression of AQP1 can be associated with increased mortality and accelerated metastatic progression in MM, assuming a possible association between AQP1 and BRAF V600E mutation [10,11,12]. Conversely, Osorio et al. [13] have found that AQP1 expression is downregulated in NMSC and melanoma compared to control skin and benign nevi. AQP8 is mainly expressed in the liver, colon, and pancreatic acinar cells [3,14], and its downregulation has been associated with reduced survival in colon adenocarcinoma patients [15]. However, so far, the correlation between AQP8 and MM development has not been studied yet. AQP9 is expressed in several tissues, including the skin where it is mainly expressed by keratinocytes of the stratum granulosum [16]. Several studies have demonstrated that AQP9 expression is altered in several tumors and inflammatory diseases, despite no information being available on AQP9’s involvement in skin cancer development. Taking in consideration the lack of literature in this field, our study aims to fill this gap through the evaluation of AQP expression and its correlation with the clinical and histopathological characteristics and the outcomes of MM patients.

## 2. Materials and Methods

### 2.1. Patient Population

This retrospective observational study was conducted on 58 patients with MM, identified among patients under follow-up at the Dermatology Department, AOU Maggiore Hospital (Novara, Italy). The subjects were selected using the available clinical folders, commonly employed in clinical practice for reporting and consulting histological examinations. The inclusion criteria were: (a) diagnosis of primary MM stage IB occurred in a period between 1 January 2014 and 31 December 2016; (b) availability of histological slides; (c) availability of histological report and clinical information. The data were reported in the REDCap platform, validated by the University of Eastern Piedmont for data collection and statistical analysis, ensuring patient anonymity. In detail, we analyzed: (a) demographic data (i.e., gender and average age at MM diagnosis); (b) MM histological features: histotype, site, presence of ulceration, Clark level, and Breslow index (for Breslow index, the patients were subcategorized into the following three groups: <2 mm, 2–4 mm, and +4 mm); (c) mitotic index; (d) mutational status (presence/absence of BRAF mutations including V600E and V600K); (e) sentinel lymph node status (sentinel lymph node biopsy, SLNB) categorized as positive, negative, not performed, and/or not found; (f) presence of lymph node metastases and systemic metastasis identified clinically or via imaging (ultrasound evaluation, US; tomography computer-based total body, TC; positron emission tomography, PET); (g) stage of disease at diagnosis of primary MM; (h) assessment of progression-free survival (PFS) up to the last follow-up date set at 31 December 2021, up to the date of death, or the last available follow-up; (i) date of death, in case of death, obtained through a formal request to the competent offices in compliance with the most current personal data protection regulation. All characteristics are shown in Table 1.

### 2.2. Immunohistochemistry

We cut 4 μm thick tissue sections from paraffin-embedded (FFPE) melanoma biopsies and stained those through immunohistochemistry (IHC). Immunohistochemical staining of AQP1 was performed using Ventana Benchmark ULTRA (Roche, Basilea, Swiss), with a mouse anti-AQP1 antibody (1:150, MA5-25401; Thermo Fisher, Waltham, MA, USA) following manufactur’s instructions. AQP8 and -9 immunohistochemical stains were performed manually. Sections were incubated for 30 min at 56 °C in order to eliminate excess water, and were dewaxed in xylene, hydrated in graded ethanol (100, 100, 95, 90 and 75%), and washed in PBS 1x. Then, slides were heated with Tris-based unmasking solution pH 9.0 in a microwave (Vector Laboratories, Burlingam, CA, USA), at a power of 550 W for 5 min, and cooled down for 30 min. Endogenous peroxidases were blocked by incubating slides for 10 min with 3% H_2_O_2_ in the dark (Merck, Darmstadt, Germany). In a humidity chamber, samples were incubated with Vectastain Universal Elite ABC kit Blocking Solution (Vector Laboratories) for 30 min at room temperature (RT). Then, mouse anti-AQP8 (1:500, MA5-27355; Thermo Fisher) and rabbit anti-AQP9 (1:100, PA5-51285; Thermo Fisher), diluted in Blocking Solution, were added to slides and incubated for 1 h at RT in a humidity chamber. Subsequently, samples were incubated with secondary antibody Vectastain Universal Elite ABC kit Biotinylated Horse and anti-mouse Ig/rabbit Ig secondary antibodies (Vector Laboratories) for 30 min at RT, rinsed in PBS, and incubated with Vectastain Universal Elite ABC reagent for 30 min at RT. Slides were washed with PBS, incubated with ImmPACT DAB Substrate (Vector Laboratories), and counterstained with hematoxylin (Bio-Optica, Milan, Italy). Then, dehydration was performed by incubating the samples with graded ethanol (75, 95, 100, 100%) and xylene for 5 min at RT. Coverslips were mounted using VectaMount Mounting Media (Vector Laboratories), and samples were visualized with Panoramic MIDI II (3DHistech, Budapest, Hungary). DAB stain evaluation was performed using the IHC Profiler [17] plugin for ImageJ Software 1.53e [18] (http://rsb.info.nih.gov/ij/ accessed on 17 October 2023). Briefly, we created a region of interest (ROI) around the lesion, and we ran the IHC Profiler plugin that automatically scores the IHC stain as “negative”, “low positive”, “positive”, or “high positive”. Since we did not obtain any “high positive” results, and considering the small number of samples analyzed, we grouped together the “low positive” and “positive” results, creating two separated groups identified as “negative” and “positive” lesions.

### 2.3. Statistical Analysis

Descriptive statistics were conducted, reporting absolute and relative frequencies for categorical variables, and mean with standard deviation or median with interquartile range for numerical ones. Analyses were reported separately for negative and positive AQP stains. Differences between groups in terms of clinical features were evaluated using the chi-square or Fisher test and *t*-test. Progression time was also calculated, and Kaplan–Meier curves were reported for all subjects and separately for negative and positive AQP stains. The difference in time was assessed using the log rank test. All the analyses were conducted using SAS 9.4 and STATA 18SE and the statistical threshold was set to 0.05 (two-tailed).

## 3. Results

### 3.1. Clinical and Immunohistochemical Characteristics of Patients

The clinical characteristics of our patients are summarized in Table 1. The sample consisted of 58 subjects, 30 (51.7%) males and 28 (48.3%) females, with a mean age of 59.7 years (SD 14.0). We found that 75.9% (*n* = 44) of melanomas were SSM and the remaining were NM; the mean Breslow thickness was 2.47 mm (SD 2.46). A BRAF mutation (V600E or V600K) was identified in 46.6% (*n* = 27) of the samples. In 20 (35.09%) patients, the SLN biopsy demonstrated the presence of macro- or micro-metastases, and 18 patients underwent lymphadenectomy, according to current guidelines.

Initially, we tested, using IHC, the expression of AQP3, AQP5, and AQP10 on a small group of both SSM and NM patients, but none of these AQPs were expressed (Supplementary Figure S1). We, therefore, focused our attention on AQP1, -8, and -9, which were found to be positively expressed in 11 (19.0%), 41 (69.0%), and 44 (77.6%) patients, respectively.

### 3.2. Immunohistochemical Expression of AQP1, -8, and -9 in Primary Melanoma

**Figure 1:**
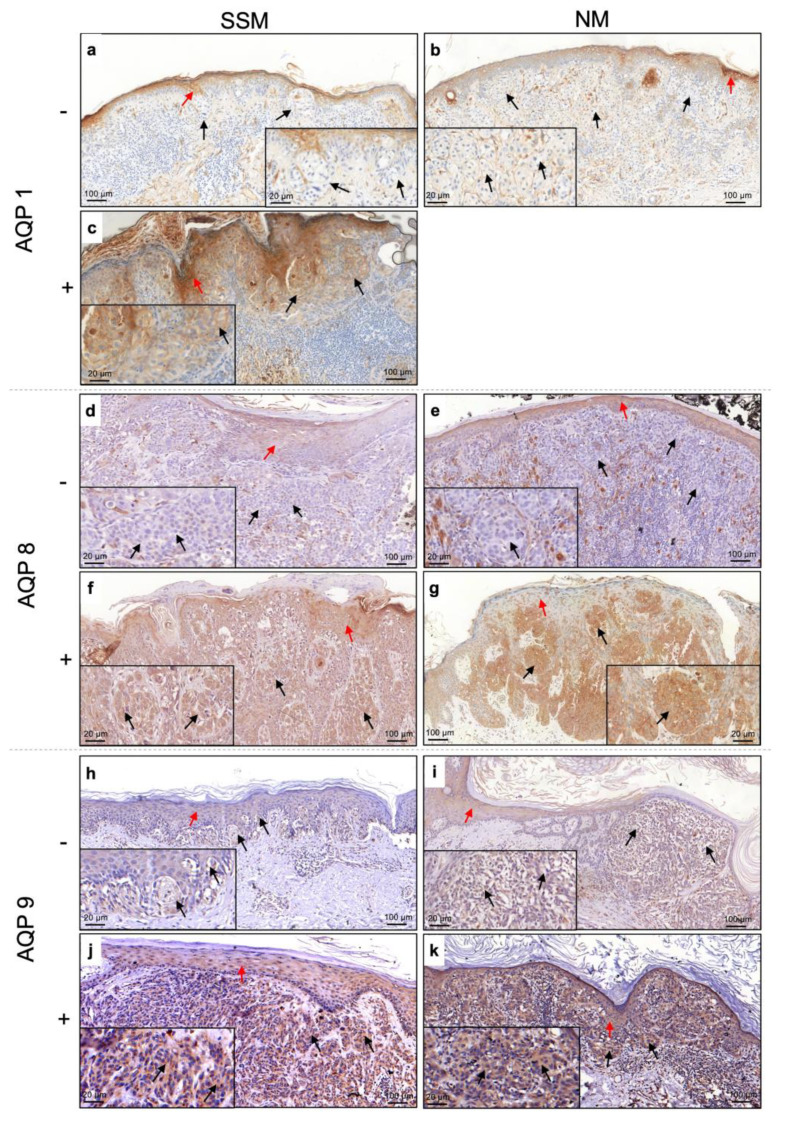
Expression of AQP1, AQP8, and AQP9 on SSM and NM (black arrow) and epidermis (red arrow). Positive stain is indicated with “+”, while negative stain is indicated with “−“. AQP1 (**a**,**b**) -negative and (**c**) -positive expression in SSM and NM; AQP8 (**d**,**e**) -negative and (**f**,**g**) -positive expression in SSM and NM; AQP9 (**h**,**i**) -negative and (**j**,**k**) -positive expression in SSM and NM. SSM, superficial spreading melanoma; NM, nodular melanoma; AQP, aquaporin.

Immunohistochemical analysis revealed that all AQPs were expressed in the cytoplasm of epidermal keratinocytes (indicated with a red arrow on Figure 1), which were considered as positive controls for the assay. Furthermore, we found that even on malignant melanocytes, AQP expression was mainly located in the cytoplasm regardless of the melanoma histotype. However, by analyzing the expression of AQPs on SSM and NM, we noted that not all lesions expressed these proteins. Indeed, we found that all NMs were negative for AQP1 expression (0/14) (Figure 1b), while the 25% of SSMs (11/44) were positive for AQP1 (Figure 1c). On the other hand, AQP8 was expressed by 77.3% (34/44) (Figure 1f) of SSM versus 50% (7/14) of NM (Figure 1g), while AQP9 was positive in 83.7% (36/43) of SSM (Figure 1j) and 57.1% (8/14) of NM (Figure 1k). However, no statistical difference was found (*p* > 0.05).

### 3.3. AQP1 Expression Is Associated with the Site of Melanoma and Mitotic Index

The expression of AQP1 was significantly associated with the body site (*p* = 0.035) and the mitotic index (*p* = 0.005). Specifically, it appeared that AQP1 was more frequently expressed in melanomas located in the upper limbs (42.9%, 6/14), followed by the torso/head/neck (14.3%, 4/28) and lower limbs (6.3%, 1/10). Moreover, we observed that AQP1 was mostly expressed in melanomas with a mitotic index ≤1 (100% vs. 10%). Results are shown in Table 2.

### 3.4. AQP8 Is Associated with Negative Sentinel Lymph Node

Similar to AQP1 results, the difference in AQP8 expression between the SSM and NM groups was not significant (*p* = 0.089) (Table 3). However, we found a significant association (*p* = 0.0016) between AQP8 expression and a negative sentinel lymph node (86.49%), which is considered a better prognostic factor.

### 3.5. AQP9 Is Associated with the Breslow Index, Ulceration, and Age

Finally, we found that AQP9 expression was positively associated with low values of Breslow thickness and the absence of ulceration (Table 4). Moreover, it seems that AQP9 was more frequently expressed in younger people (56.1 vs. 72.1 years, *p* = 0.002). Again, no significant differences were found between the SSM and NM groups (*p* = 0.064).

### 3.6. AQP Expression Improves the Disease-Free Survival

**Figure 2:**
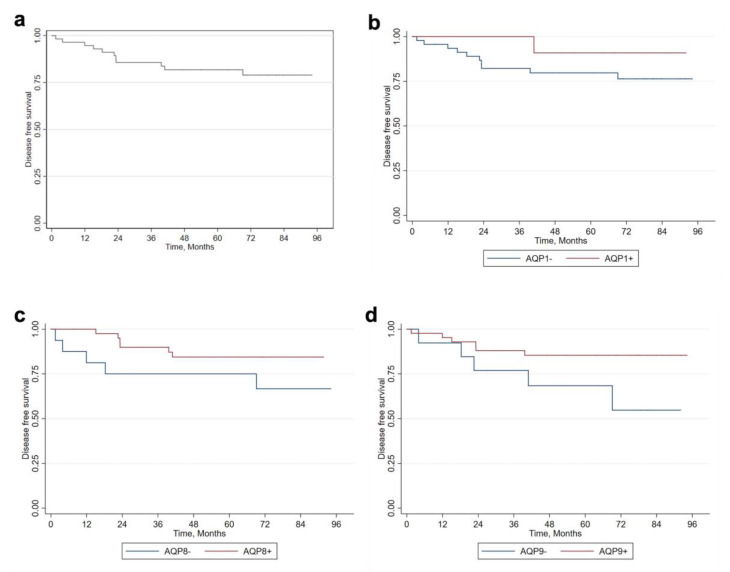
Analysis of disease-free survival based on AQP expression. (**a**) Disease-free survival of melanoma patients. Kaplan–Meier survival curve based on (**b**) AQP1, (**c**) AQP8, and (**d**) AQP9 expression. AQP, aquaporin.

Lastly, the association between AQP expression and disease-free survival is reported in Figure 2. In general, over the 8-year survival period, approximately 75% of patients, maintained freedom from the disease, suggesting that patients that positively expressed AQPs had a better prognosis. Indeed, even though all differences were not statistically significant, we found that only 9.09% of patients positive for AQP1 registered cancer progression, compared to 21.28% among negative patients (*p* = 0.67). Similarly, among AQP8-positive patients, only 14.63% had melanoma progression, which was lower compared to the negative group (29.41%, *p* = 0.270). Finally, 13.64% of AQP9-positive patients registered melanoma progression, which was lower compared to AQP9-negative patients, who showed a higher progression rate of 38.46% (*p* = 0.1023).

## 4. Discussion

Several in vivo and in vitro studies have demonstrated that tumorigenesis can be influenced by the expression of AQPs. In fact, these proteins are involved in migratory and metastasizing capacities of tumor cells, in the stimulation of neoangiogenesis, and in the epithelial–mesenchymal transition [19]. The most important isoform implicated in this process is AQP1, which is expressed in peripheral vascular endothelial cells, can be upregulated by pro-angiogenic factors in response to hypoxia, is essential for the migration of endothelial cells and for tumoral angiogenesis [20].

Many tumors, such as lung cancer, colon cancer, HCC, and glioblastoma, have aberrant expression of AQPs [21], variably associated with prognosis. To date, the two most studied isoforms in skin cancers are AQP1 and AQP3 [22,23], while scarce or missing data concern the association of AQP8 and AQP9 with MM. Also, changes in the AQP expression during processes of neoplastic transformation need to be clarified: our preliminary results on a small sample of melanocytic nevi confirmed the expression of AQP1, in agreement with those previously reported by literature [13], whereas data for AQP8 and AQP9 need to be studied on a larger sample.

Our study aimed to fill this gap, evaluating the possible prognostic value of AQPs, by retrospectively analyzing their expression in a series of 58 melanoma patients. The epidemiological, clinical, and histopathological characteristics of our cases make our study comparable with those published so far in the literature [11,24].

In our experience, AQP expression has mainly been histotype-dependent. For instance, AQP1 was exclusively expressed by SSM and absent in all NM cases. Similarly, the number of AQP8- and AQP9-positive cases was higher among SSM than NM. Even if the relatively small number of cases examined does not allow statistical significance, this first observation suggests a potential correlation with better prognosis, in consideration of the different clinical courses of the two main histological types of melanomas [25]. Moreover, we demonstrated a significative association between the expression of AQP1, the body site, and the mitotic index. The low mitotic index is a widely confirmed favorable prognostic factor for melanoma patients [26], as well as the localization to the limbs, which slows down the possibility of visceral spreading compared to the torso [27,28].

Regarding AQP8, we found a significant association between its expression and a negative sentinel lymph node, which is considered an advantageous prognostic factor. Moreover, AQP9 expression was positively associated with low Breslow thickness values and the absence of ulceration, also known as favorable prognostic factors [28].

In our case, it was not possible to verify a significant correlation between the expression of AQP1, -8, and -9 and mutational status. BRAFWT melanomas were found in 53.45% of our sample, whereas the remaining 46.55% had BRAFMUT (V600E and V600K). No statistically significant differences were found concerning the expression of BRAF, unlike reports by other authors, who hypothesized that the constitutive activation of the ERK pathway (observed in MC cells carrying the BRAFV600 mutation) can be responsible for the increased expression of AQP1 in patients with advanced disease [11]. In the study by Imredi et al. [11], 44.28% of patients had BRAFMUT and the majority of these belonged to late-stage disease (III–IV). The presence of BRAFV600 was correlated to a significantly higher expression of AQP1 in the endothelium (*p* = 0.014).

The possible favorable role of AQPs in the prognosis of patients with melanoma is also supported by the survival analyses conducted in our study. The 8-year overall survival rate was around 75%, with a better prognosis in AQP-positive patients. Indeed, we found that only 9.09% of patients positive for AQP1 registered cancer progression compared to negative patients, who reached 21.28% (*p* = 0.67). Similarly, among the AQP8-positive patients, only 14.63% had melanoma progression compared to the negative group (29.41%, *p* = 0.270). Finally, 13.64% of AQP9-positive patients registered melanoma progression compared to 38.46% of AQP9-negative patients (*p* = 0.1023). No differences were statistically significant, and only further studies on larger samples will be able to confirm the validity of these results.

## 5. Conclusions

Overall, our work highlights new concepts in terms of correlation between AQP expression, clinical and histopathological characteristics, and disease course in melanoma, as well as the opportunity to target them for future melanoma therapies. A limitation of our study is represented by the relatively low sample size, which may have affected the statistical significance of some results, including the possibility of identifying a correlation between the expression of AQPs and the level of differentiation. Considering the possible relationship between AQPs and dysplasia in melanocytic lesions and the scarcity of literature on this topic, a possible expansion of our work is represented by the study of a large panel of skin lesions, to confirm their possible diagnostic/prognostic value.

## Figures and Tables

**Table 1:** Clinical characteristics of patients enrolled in this study.

	*n* (%)
**Gender**	
Male	30 (51.72)
Female	28 (48.28)
**Age, years**	
**Mean (SD)**	59.72 (13.98)
**Median [Q1–Q3]**	59 [50; 72]
**Sentinel lymph node**	20 (35.09)
**Lymphadenectomy**	18 (31.03)
**Melanoma relapse**	6 (10.34)
**History of melanoma**	3 (5.17)
	**Melanoma characteristics**
**Histotype**	
Superficial spreading melanoma (SSM)	44 (75.86)
Nodular melanoma (NM)	14 (24.14)
**Site**	
Head/neck	1 (1.72)
Torso	27 (46.55)
Upper limbs	14 (24.14)
Lower limbs	16 (27.59)
**Breslow**	
Mean (SD)	2.47 (2.46)
Median [Q1–Q3]	1.53 [0.83; 2.70]
<2	32 (55.17)
<2; 2–4; >4	16 (27.59)
4+	10 (17.24)
**Ulceration**	10 (17.24)
**Clark level**	
II	7 (12.07)
III	15 (25.86)
IV	36 (62.07)
**Mitotic index**	
0	3 (5.17)
1+	55 (94.83)
Mean (SD)	4.07 (4.62)
Median [Q1–Q3]	2 [1; 6]
**BRAF mutation**	27 (46.55)
V600E	21 (77.8)
V600K	6 (22.2)

**Table 2:** Statistical analysis of possible correlation between AQP1 expression and clinical characteristics. * Indicates statistical significant *p* values.

		AQP1		
**Variable**		Negative	Positive	*p*-value
	Number (%)	47 (81.03)	11 (18.97)	
**Gender**				
	Male	23 (76.67)	7 (23.33)	0.5079
	Female	24 (85.71)	4 (14.29)	
**Age, years**				
	Mean (SD)	59.55 (13.98)	60.45 (14.60)	0.8493
**Histotype**				
	SSM	33 (75.00)	11 (25.00)	0.0502
	NM	14 (100)	0	
**Site**				
	Trunk	24 (85.71)	4 (14.29)	0.0348 *
	Upper limb	8 (57.14)	6 (42.86)	
	Lower limb	15 (93.75)	1 (6.25)	
**Breslow**				
	<2	23 (71.88)	9 (28.13)	0.1792
	2–4	15 (93.75)	1 (6.25)	
	4+	9 (90.00)	1 (10.00)	
**Clark**				
	II	4 (57.14)	3 (42.86)	0.1883
	III	12 (80.00)	3 (20.00)	
	IV	31 (86.11)	5 (13.89)	
**Ulceration**				
	No	38 (79.17)	10 (20.83)	0.6685
	Yes	9 (90.00)	1 (10.00)	
**Mitotic index**				
	0	0	3 (100)	0.0054 *
	>1	47 (85.45)	8 (14.55)	
**BRAF mutation**				
	Negative	25 (80.65)	6 (19.35)	>0.999
	Positive	22 (81.48)	5 (18.52)	
**Sentinel lymph node**				
	Negative	29 (78.38)	8 (21.62)	0.4674
	Positive	18 (90.00)	2 (10.00)	

**Table 3:** Statistical analysis of the correlation between AQP8 expression and melanoma. * Indicates statistical significant *p* values.

		AQP8		
**Variable**		Negative	Positive	*p*-value
	Number (%)	17 (31.03)	41 (68.97)	
**Gender**				
	Male	11 (36.67)	19 (63.33)	0.2550
	Female	6 (21.43)	22 (78.57)	
**Age, years**				
	Mean (SD)	65.12 (13.62)	57.49 (13.66)	0.0577
**Histotype**				
	SSM	10 (22.73)	34 (77.27)	0.0889
	NM	7 (50.00)	7 (50.00)	
**Site**				
	Trunk	10 (35.71)	18 (64.29)	0.5251
	Upper limb	4 (28.57)	10 (71.43)	
	Lower limb	3 (18.75)	13 (81.25)	
**Breslow**				
	<2	6 (18.75)	26 (81.25)	0.1026
	2–4	6 (37.50)	10 (62.50)	
	4+	5 (50.00)	5 (50.00)	
**Clark**				
	II	1 (14.29)	6 (85.71)	0.3915
	III	3 (20.00)	12 (80.00)	
	IV	13 (36.11)	23 (63.89)	
**Ulceration**				
	No	13 (27.08)	35 (72.92)	0.4580
	Yes	4 (40.00)	6 (60.00)	
**Mitotic index**				
	0	0	3 (100)	0.5482
	>1	17 (30.91)	38 (69.09)	
**BRAF mutation**				
	Negative	8 (25.81)	23 (74.19)	0.5741
	Positive	9 (33.33)	18 (66.67)	
**Sentinel lymph node**				
	Negative	5 (13.51)	32 (86.49)	0.0016 *
	Positive	11 (55.00)	9 (45.00)	

**Table 4:** Statistical analysis of possible correlation between AQP9 expression and melanoma characteristics. * Indicates statistical significant *p* values.

		AQP9		
**Variable**		Negative	Positive	*p*-value
	Number (%)	13 (22.41)	44 (77.59)	
**Gender**				
	Male	10 (34.48)	19 (65.52)	0.0563
	Female	3 (10.71)	25 (89.29)	
**Age, years**				
	Mean (SD)	72.08 (10.05)	56.05 (13.07)	0.0002 *
**Histotype**				
	SSM	7 (16.28)	36 (83.72)	0.0643
	NM	6 (42.86)	8 (57.14)	
**Site**				
	Trunk	9 (32.14)	19 (67.86)	0.1948
	Upper limb	1 (7.14)	13 (92.86)	
	Lower limb	3 (20.00)	12 (80.00)	
**Breslow**				
	<2	1 (3.23)	30 (96.77)	0.0001 *
	2–4	8 (50.00)	8 (50.00)	
	4+	4 (40.00)	6 (60.00)	
**Clark**				
	II	1 (14.29)	6 (85.71)	>0.9999
	III	4 (26.67)	11 (73.33)	
	IV	8 (22.86)	27 (77.14)	
**Ulceration**				
	No	7 (14.89)	40 (85.11)	0.0060 *
	Yes	6 (60.00)	4 (40.00)	
**Mitotic index**				
	0	0	3 (100)	>0.9999
	>1	13 (24.07)	41 (75.93)	
**BRAF mutation**				
	Negative	8 (26.67)	22 (73.33)	0.5385
	Positive	5 (18.52)	22 (81.48)	
**Sentinel lymph node**				
	Negative	6 (16.22)	31 (83.78)	0.1036
	Positive	7 (36.84)	12 (63.16)	

## Data Availability

The data are not publicly available.

## Supplementary Materials

The following supporting information can be downloaded at: https://www.mdpi.com/article/10.3390/jcm12227137/s1. Figure S1: Immunohistochemical stain of AQP3, 5 and 10 on primary melanomas.
