# Supplementary material for: Aquaporin 1, Aquaporin 8, and Aquaporin 9 Expressions in Malignant Melanoma: A Possible Correlation with Prognosis and Clinical Outcome
Source: J Clin Med. 2023 Nov 16;12(22):7137. doi: 10.3390/jcm12227137 (PMC10672695; doi:10.3390/jcm12227137)
Supplement: Supplementary file 1 [file jcm-12-07137-s001.zip › jcm-2697489-supplementary.pdf]

Supplementary materials

Supplementary Figure S1.

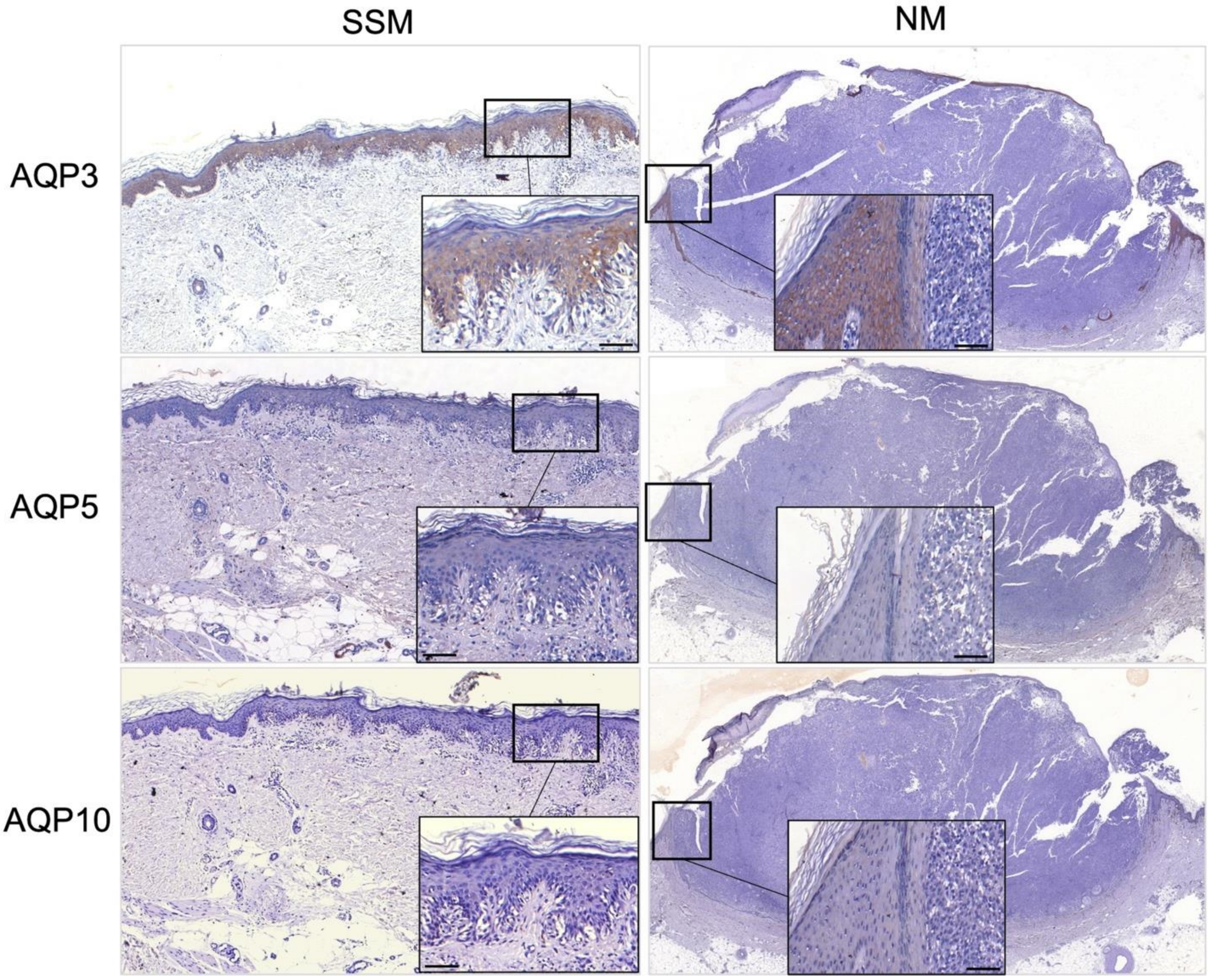

Figure S1: Immunohistochemical stain of AQP3, 5 and 10 on primary melanomas. Representative IHC pictures of SSM and NM stained for AQP3, 5 and 10. Scale bar: 50 µm. SSM, superficial spreading melanoma; NM, nodular melanoma; AQP, aquaporin.
